# HIV–Cholesterol Connection Suggests a New Antiretroviral Strategy

**DOI:** 10.1371/journal.pbio.0040400

**Published:** 2006-10-31

**Authors:** Liza Gross

Straddling the line between living and nonliving, a virus must commandeer the molecular machinery of the cell it infects to persist, and sometimes even alters the cellular environment toward that end. The HIV genome encodes nine proteins, some playing multiple roles to help the virus invade human immune cells and co-opt cellular proteins—and even normal cellular activities—to reproduce. For example, a recent study showed that an HIV protein called Nef, which is required for viral replication and infection, also binds cholesterol and delivers it to the cell membrane where new virus particles (called virions) are assembled. Once assembled, virions take part of the lipid-rich cell membrane with them as they bud off in search of new cells to infect.

Now, in a new study, Zahedi Mujawar, Michael Bukrinksy, Dmitri Sviridov, and colleagues show that the HIV Nef also disrupts cholesterol trafficking machinery in the macrophages infected by the virus. By preventing the normal efflux of cholesterol from macrophages, Nef ensures that nascent virions have access to a steady supply of cholesterol. Unfortunately, what’s good for the virus causes even more trouble for a person infected with HIV: reduced cholesterol efflux from macrophages may explain why patients with HIV face increased risk of atherosclerosis and coronary artery disease.

Normally, excess cholesterol is exported to molecules in the extracellular space. The authors found that cholesterol efflux to an extracellular molecule called apoA-I was impaired in HIV-infected macrophages. Lower levels of efflux correlated with higher levels of viral replication. To see whether Nef might affect efflux impairment, the authors infected macrophages with HIV strains carrying either a functional or mutated *Nef* gene (known to affect cholesterol binding and delivery). Cholesterol efflux to apoA-I was “substantially reduced” in cells infected with functional Nef. In contrast, cholesterol efflux in cells infected with Nef-deficient strains was similar to that seen in uninfected cells—allowing the authors to conclude that HIV needs Nef to block cholesterol efflux.

Cholesterol is removed from macrophages by proteins called ATP-binding cassette (ABC) transporters, which hand off their cargo to different molecules. Having shown that Nef hinders efflux to apoA-I, the authors reasoned that Nef does so by targeting apoA-I’s donor—ABCA1. This conclusion is supported by two lines of evidence: stimulating expression of ABCA1 in infected cells significantly reduced Nef-mediated impairment of efflux to ApoA-I, but efflux was unaffected when ABCA1 levels were very low. Nef mediates these effects, the authors show, by specifically down-regulating ABCA1 after transcription and limiting its abundance, and by keeping ABCA1 sequestered at the membrane. Whether these mechanisms work in tandem or operate independently is a question for future study.

**Figure:**
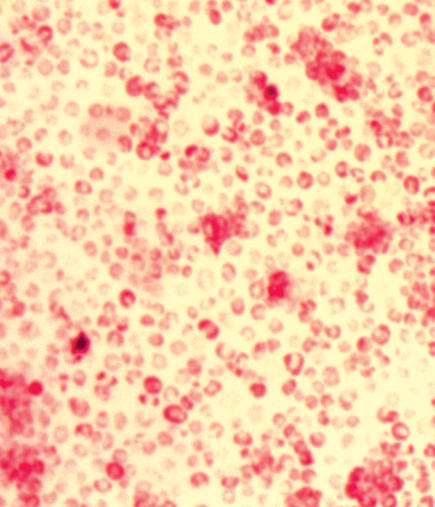
HIV-infected monocyte-derived macrophages show lipid accumulation (lipids are stained red) in HIV-infected cells. (Image: Zahedi Mujawar)

HIV-infected macrophages that expressed Nef not only contained far more lipid-containing vacuoles than uninfected cells or cells infected with Nef-deficient HIV, they also synthesized molecules associated with cholesterol accumulation and cholesterol-laden “foam cells.” And when the authors analyzed atherosclerotic plaque sections taken from patients with HIV who had been treated with anti-retroviral therapy (HAART), they found HIV-infected, cholesterol-laden macrophages in the atherosclerotic plaque—suggesting that these cells contribute to arterial disease.

Altogether, the results show that HIV inhibits cholesterol efflux through Nef, leading to cholesterol accumulation and foam cell formation. They also suggest potential targets for controlling both HIV and atherosclerosis. When the authors increased ABCA1 levels to stimulate cholesterol efflux—having observed a link between reduced efflux and increased viral replication—the treatment restored efflux and reduced virion infectivity by reducing virion incorporation of cholesterol. This suggests that stimulating cholesterol efflux may not only reduce the risk of atherosclerosis but may also help control HIV replication in HIV-infected patients.

